# Tumor-infiltrating CD8+ and FOXP3+ lymphocytes before and after neoadjuvant chemotherapy in cervical cancer

**DOI:** 10.1186/s13000-018-0770-4

**Published:** 2018-11-24

**Authors:** Yun Liang, Weiguo Lü, Xiaofei Zhang, Bingjian Lü

**Affiliations:** 1 Department of Surgical Pathology, Women’s Hospital, School of Medicine, Zhejiang University, Province, Zhejiang, 310006 China; 2 Center for Uterine Cancer Diagnosis & Therapy of Zhejiang Province, Women’s Hospital, School of Medicine, Zhejiang University, Province, Zhejiang, 310006 China; 3 Department of Gynecologic Oncology, Women’s Hospital, School of Medicine, Zhejiang University, Province, Zhejiang, 310006 China

**Keywords:** Squamous cell carcinoma, Cervix, Tumor infiltrating lymphocytes, Neoadjuvant chemotherapy

## Abstract

**Background:**

Neoadjuvant chemotherapy (NACT) has been recently accepted as an effective alternative in patients with locally advanced cervical cancer. However, little is known about the effects of NACT on the immunological microenvironment in cervical cancers. In this study, we analyzed the alterations of tumor infiltrating lymphocytes (TILs) before and after NACT and analyzed their prognostic significance in advanced cervical cancer patients treated with platinum-based NACT.

**Methods:**

We recruited 137 patients with stage Ib2 and IIa2 cervical cancer retrospectively. Pretreatment biopsy and surgical specimens after NACT were immunostained with CD8 and Foxp3. The densities of intratumoral and peritumoral immunopositive TILs were analyzed separately.

**Results:**

Foxp3+ T cells density significantly decreased in both intratumoral (median 28.49 vs. 19.97; Z = − 8.635, *p* < 0.001) and peritumoral (median 113.53 vs. 82.48; Z = − 3.741, *p* < 0.001) areas after NACT, whereas CD8+ T cell counts remained stable in both intratumoral (median 121.32 vs. 109.59; Z = − 0.817,*p* = 0.414) and peritumoral (median 402.56 vs. 390.84; Z = − 1.138,*p* = 0.255) areas. Patients with pathological complete response (pCR) had significantly lower number of Foxp3+ T cell density after NACT than non-pCR cases in both intratumoral (median16.12 vs. 22.00; Z = − 2.009, *p* = 0.045) and peritumoral areas(median 63.31 vs. 98.48; Z = − 2.469, *p* = 0.014). Multivariate analyses demonstrated that high ratio of intratumoral CD8/peritumoral Foxp3 in residual tumors was independent prognostic factor for both progression-free survival (HR = 0.297; 95% CI, 0.109–0.810, *p* = 0.018) and overall survival (HR = 0.078; 95% CI, 0.010–0.598, *p* = 0.014).

**Conclusions:**

NACT in cervical cancers can induce anti-cancer immunity by altering TILs subsets. An elevated intratumoral CD8/peritumoral Foxp3 ratio after NACT may confer a favorable clinical outcome.

## Background

The interaction between tumor cells and microenvironment plays an essential role in the progression of malignant tumors. The major component of tumor microenvironment is tumor infiltrating lymphocyte (TIL), a selected population of T-cells with a higher specific immunological reactivity against tumor cells. Cervical cancer is the second most common cancer in women worldwide and is initiated by persistent infection of high risk human papillomatous virus (HPV) [1]. The immune microenvironment in cervical cancer not only shares the common features in solid tumors, but also harbors the unique properties that are associated with HPV infection [2].

Neoadjuvant chemotherapy (NACT) has emerged as a promising method in the management of locally advanced cervical cancer. Systemic chemotherapy may kill cancer cells by direct cytotoxicity, and through the immune system as suggested by recent data [3, 4]. The complex network of immune cells in tumor microenvironment can influence the effects of anticancer treatment.

For locally advanced cervical cancer, although the histopathologic alterations following NACT has been reported recently [5], the effects of NACT on the interactions between tumor and host in situ have not well been investigated so far. Comparative analysis of pre-therapeutic biopsy and the post-chemotherapeutic removed tumors provides an opportunity to assess the influence of chemotherapy on local immune microenvironment. In the present study, we observed the dynamic changes of CD8+ and Foxp3+ T cells in cervical cancer patients with NACT. Our specific aim is to assess the prognostic value of these immune cells.

## Methods

### Patient selection and clinical information

In this retrospective study, we consecutively recruited 137 patients with bulky FIGO stage Ib2 and IIa cervical squamous cell carcinoma between January 2007 and December 2014 in Women’s Hospital School of Medicine Zhejiang University, China (Table 1). The study was approved by the Medical Ethics Committee of the hospital, and all patients or their relatives were consent with this study in written forms. The patients were histologically confirmed before NACT by cervical biopsy under colposcopy. After 3–4 weeks following 2 cycles of TP chemotherapy (paclitaxel175 mg/m^2^ [3-h infusion] + cisplatin 75 mg/m^2^), the patients underwent a radical hysterectomy. Post-operative radiotherapy, chemotherapy or concurrent chemo-radiation therapy was carried out in patients with a positive surgical margin, paracervical involvement, deep stromal invasion, lymph node metastasis or vascular invasion.

**Table 1:** Patient characteristics

Characteristic		*n*	(%)
FIGO stage	Ib2	91	66.4
	IIa2	46	33.6
Histological grade	I-II	109	79.6
	III	28	20.4
Lymph node metastasis	Yes	20	14.6
	No	117	85.4
Chemotherapy regimen	BVP	32	23.4
	TP	105	76.6
Clinical response	Response	101	73.7
	Non-response	36	26.3
Optimal pathologic response	OR	25	18.2
	Non-OR	112	81.8

Tissue samples were immediately fixed in 10% neutral buffered formalin and embedded in paraffin. Clinical response was evaluated by vaginal pelvic examination and abdominal/pelvic CT scan before and after chemotherapy*.* The World Health Organization (WHO) clinical tumor response criteria was applied to define complete response (CR) as the disappearance of all known tumors, partial response (PR) as 50% or more decrease in the total tumor volume, no change (NC) as a less than 50% decrease in total tumor or has a less than 25% increase in the size of measurable lesions, and progressive disease (PD) as a 25% or more increase in the size of measurable lesions [6]. CR or PR is regarded as clinically effective, and NC or PD as non-effective. Pathological complete response (pCR) to NACT was defined as complete disappearance of tumor, residual disease with < 3 mm stromal invasion with negative lymph nodes, or carcinoma in situ [7]. The patients have been followed up for 51 months on an average (22–117 months).

### Immunohistochemical staining and quantification of CD8+ and Foxp3+ T cells

The expression of CD8+ and Foxp3+ T cells was analyzed by immunohistochemistry on formalin-fixed, paraffin-embedded tumor sections using a standard procedure. The primary antibodies were mouse monoclonal anti-human CD8 (DAKO Cytomation, Glostrup, Denmark;1:100 in dilution) and Foxp3 (Abcam, Cambridge, UK;1:50 in dilution). Briefly, the slides were deparaffinzed in xylene and rehydrated in graded concentrations of ethanol and distilled water. Endogenous peroxidase activity was blocked by submersion of the sections in a 0.5% H_2_O_2_/methanol solution for 10 min at room temperature. Antigen was retrieved in 10 mM sodium citrate buffer (PH 6.0) in a stainless steel pressure cooker for 1 min and 30 s after boiling, and cooled at room temperature. The slides were incubated with primary antibodies at 4 °C overnight and then incubated with DAKO EnVision for 30 min. Specific antigen-antibody reactions were visualized using 0.2% diaminobenzidine tetrahydrochloride and hydrogen peroxide.

CD8+ and Foxp3+ T cells were calculated in 10 high-power fields (five tumor beds and five peripheries) of highest density. For the Field Number (F.N.) of the eyepiece was 22 mm, and the Field Of View (FOV) was 0.237 mm^2^ for high power microscope(× 40 objective lens). The numbers per square millimeter was obtained through dividing the counts in each high-power field by the FOV. The average counts of CD8+ and Foxp3+ T cells within or peripheral to tumor were recorded separately. The number of lymphocytes were calculated and confirmed by two investigators both of whom were blinded to the clinicopathologic characteristics.

### Statistical analysis

The median value of CD8+ and Foxp3+ T cells was used to defining the cutoff of subgroups. The Wilcoxon signed-rank test and Mann–Whitney U test were applied to compare lymphocytes between different tissue locations as well as in different subgroups. Spearman’s Rank-Correlation test was applied to assess the relationship between lymphocytic variables and clinicopathologic characteristics. Univariate and multivariate logistic regression models were used to determine whether the lymphocytic variables before chemotherapy were associated with clinical chemotherapy sensitivity. Cumulative survival time was calculated by the Kaplan-Meier method. Multivariate analysis was based on the Cox proportional hazards regression model. Differences between groups were considered statistically significant if *p* < 0.05. The analysis was performed with SPSS 18.0 (SPSS Inc., Chicago, IL, USA).

## Results

### Tumor infiltrating CD8+ and Foxp3+ T cells before NACT

**Fig. 1:**
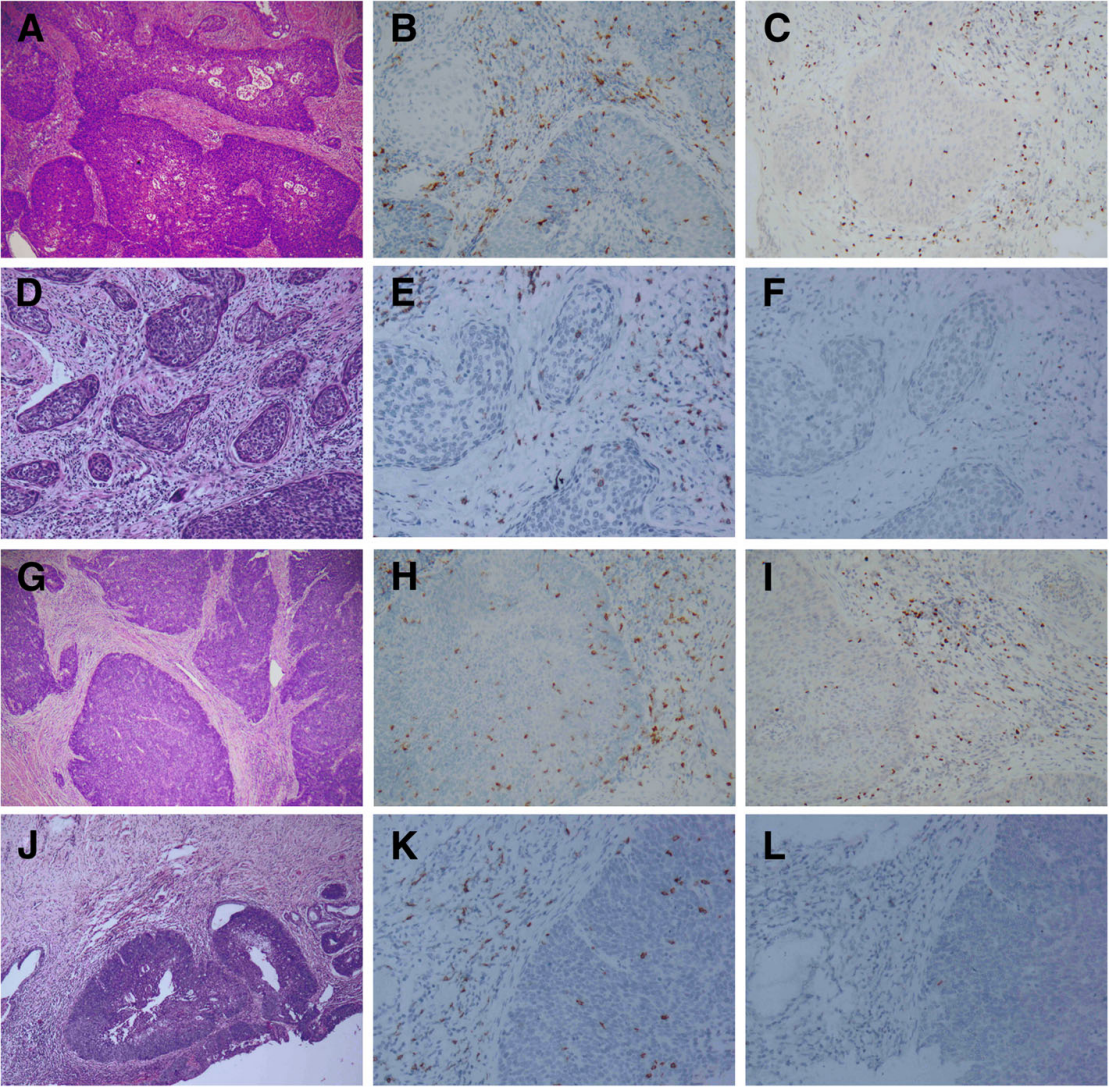
CD8+ and Foxp3+ T cells in cervical cancer before and after NACT. A pre-chemotherapy biopsy showed a typical morphology of squamous cell carcinoma (**a**). The tumor had higher CD8+ T cells (**b**) and Foxp3+ cells (**c**) in the surrounding tissue than those in the tumor nests. After NACT, the tumor didn’t achieve pathological complete response, which harbors sparse tumor nests in the stroma (**d**). Foxp3+ T cells decreased significantly (**e**), while CD8+ cells remained stable (**f**). In another case with pathological complete response, pre-chemotherapy biopsy was characteristic of squamous cell carcinoma (**g**). Both CD8+ T cells (**h**) and Foxp3+ cells (**i**) were higher in the surrounding tissue than those in the tumor nests. After NACT, the tumor only had a component of residue carcinoma in situ (**j**). CD8+ cells were not significantly changed (**k**), while Foxp3+ T cells were almost undetectable (**l**)

Membranous CD8+ and nuclear Foxp3+ T cells showed a diffuse infiltration, but not in a form of aggregates. Significantly higher density of CD8+ cells (median 402.56 vs. 121.32; Z = − 10.0411, *p* < 0.0001) and Foxp3+ T cells (median 113.53 vs. 28.49; Z = − 10.0969, *p* < 0.0001) were found in the surrounding tissues than those within the tumor nests. Representative figures are shown in Fig.1a-c, g-i.

### Relationship between CD8+, Foxp3+ T cells and clinical parameters before NACT

CD8+ and Foxp3+ T cells were categorized as high or low density in relation to their medians. High density of intra-tumoral CD8+ T cells before NACT was inversely correlated with positive lymph nodes (*r* = − 0.198, *p* = 0.021). No significant associations were identified between Foxp3+ T lymphocytes and patient age, chemotherapy regimen, FIGO stage, lymph node status or pathological grade.

### The value of tumor infiltrating T cells before NACT in prediction of clinical response to NACT

Univariate analysis indicated that high peritumoral CD8+ T cells (*p* = 0.048; OR = 2.080, 95% CI: 1.005–4.305), low peritumoral Foxp3+ T cells (*p* = 0.015; OR = 0.400, 95% CI: 0.192–0.834), together with FIGO stage (*p* = 0.083; OR = 0.520, 95% CI: 0.248–1.089), lymph node metastases (*p* = 0.098; OR = 0.444, 95% CI: 0.170–1.161) were significantly correlated with clinical effectiveness. However, multivariate analysis implicated that only low peritumoral Foxp3+ T cells (*p* = 0.020; OR = 0.406, 95% CI: 0.190–0.869) was an independent predictive factor for clinical effectiveness.

### Change of tumor infiltrating T cells after NACT and the relationship with pathological tumor response

**Fig. 2:**
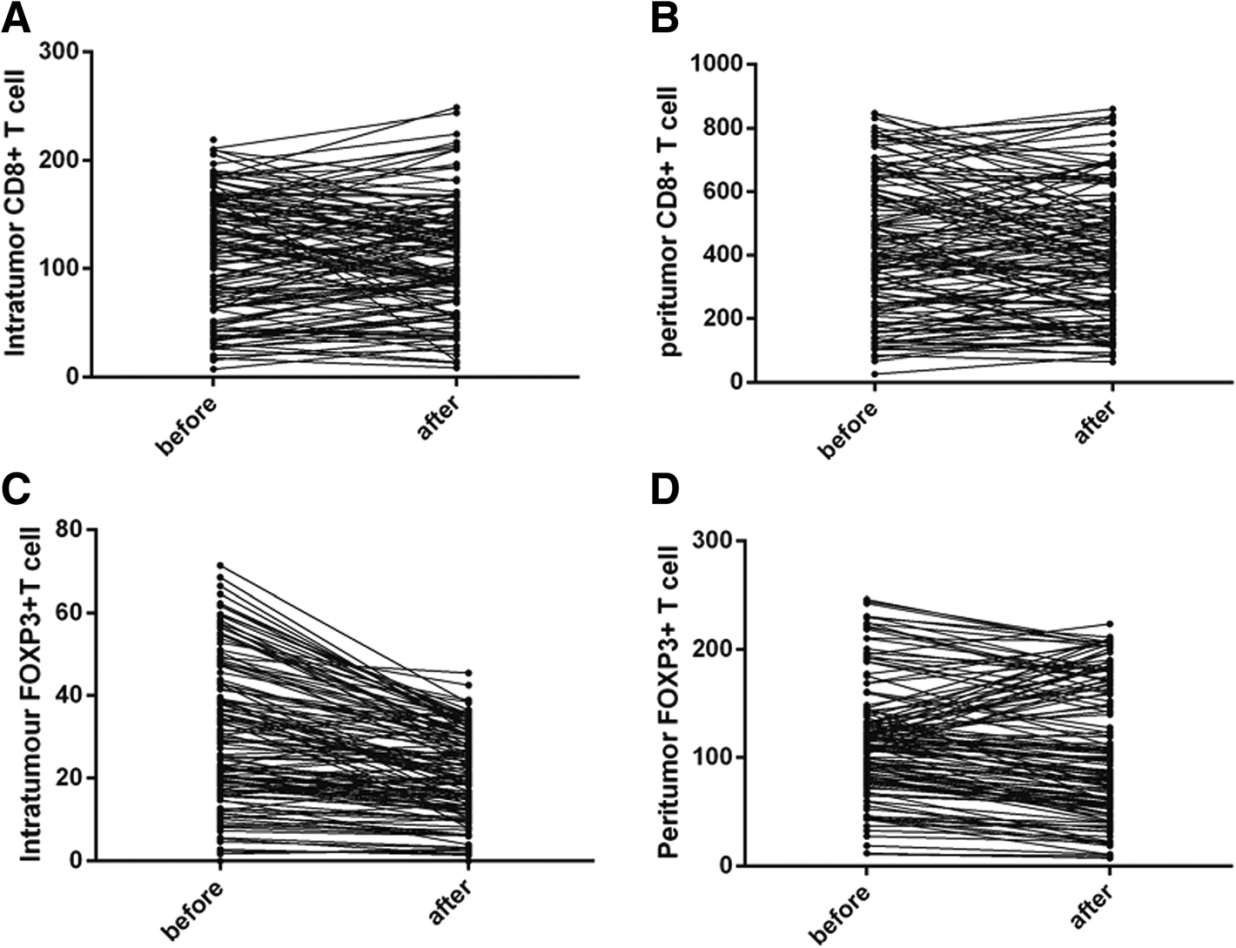
The change of CD8+ and Foxp3+ T cells in cervical cancer after NACT. CD8+ T cells did not significantly change in either intratumoral (**a**) or peritumoral (**b**) areas whereas the infiltration of Foxp3+ T cells significantly decreased in both intratumoral (**c**) and peritumoral areas (**d**) after NACT

**Fig. 3:**
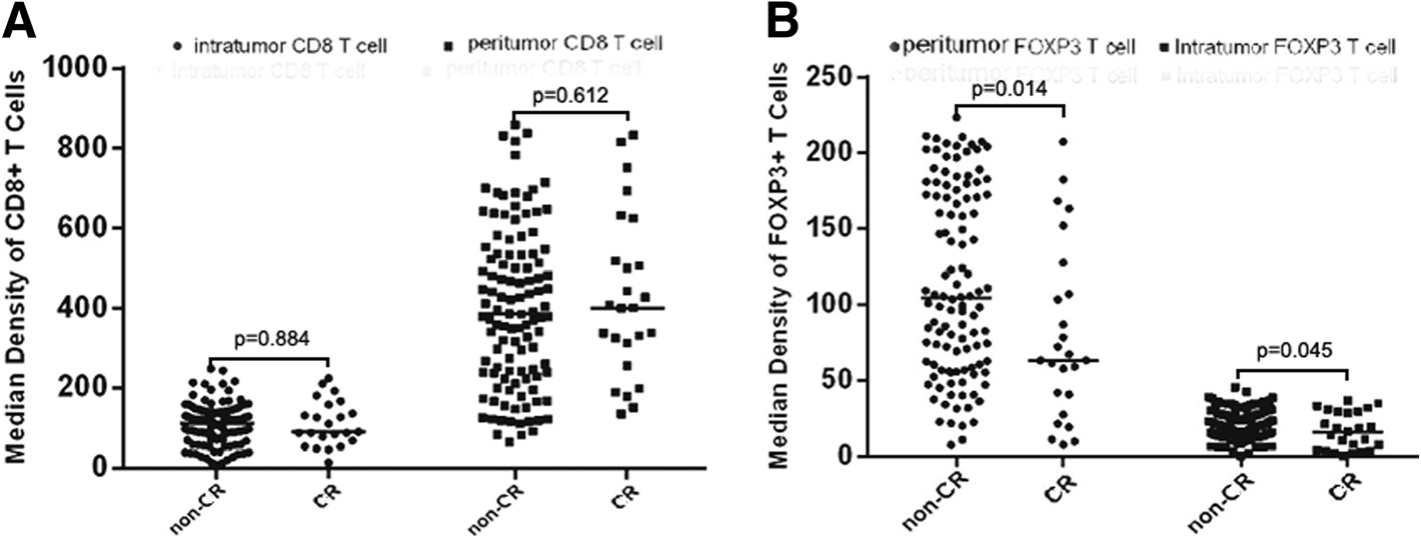
The comparison of CD8+ and Foxp3+ T cells in residual tumors between pCR and non-pCR patients. There were no significant differences of intratumoral and peritumoral CD8+ cells between pCR and non-pCR cases (**a**). However, the Foxp3+ T cells, in both intratumoral and peritumoral areas, were significantly lower in pCR patients than in non-pCR (**b**)

Comparing to the Foxp3+ T cells at biopsy specimens, post NACT Foxp3+ T cells significantly decreased in both intratumoral (median 28.49 vs. 19.97; Z = − 8.635, *p* < 0.001, Wiloxon Signed Ranks) and peritumoral (median 113.53 vs. 82.48;Z = − 3.741, *p* < 0.0001, Wiloxon Signed Ranks) areas. In contrast, the infiltration of CD8 + T cells did not significantly change after NACT in both intratumoral (median 121.32 vs. 109.59; Z = − 0.817, *p* = 0.414, Wiloxon Signed Ranks) and peritumoral (median 402.56 vs. 390.84; Z = − 1.138, *p* = 0.255, Wiloxon Signed Ranks) areas. (Fig. 1d-f, j-l; Fig. 2) implicating that Foxp3+ T cells were sensitive to chemotherapeutic effect. We further compared the numbers of CD8+ and Foxp3+ T cells between pCR and non-pCR cases in residual tumors after NACT. The patients who achieved pCR had significantly lower Foxp3+ T cells in both intratumoral (median16.12 vs. 22.00; Z = − 2.009, *p* = 0.045) and peritumoral areas (median 63.31 vs. 98.48; Z = − 2.469, *p* = 0.014, Mann-Whitney U). There were no significant differences of intratumoral (median 95.13 vs. 107.49; Z = − 0.146, *p* = 0.884, Mann-Whitney U) and peritumoral (median 386.01 vs. 401.39 Z = − 0.507, *p* = 0.612, Mann-Whitney U) CD8+ cells between pCR and non-pCR cases (Fig. 3).

### Prognostic significance of tumor infiltrating T cells after chemotherapy

**Fig. 4:**
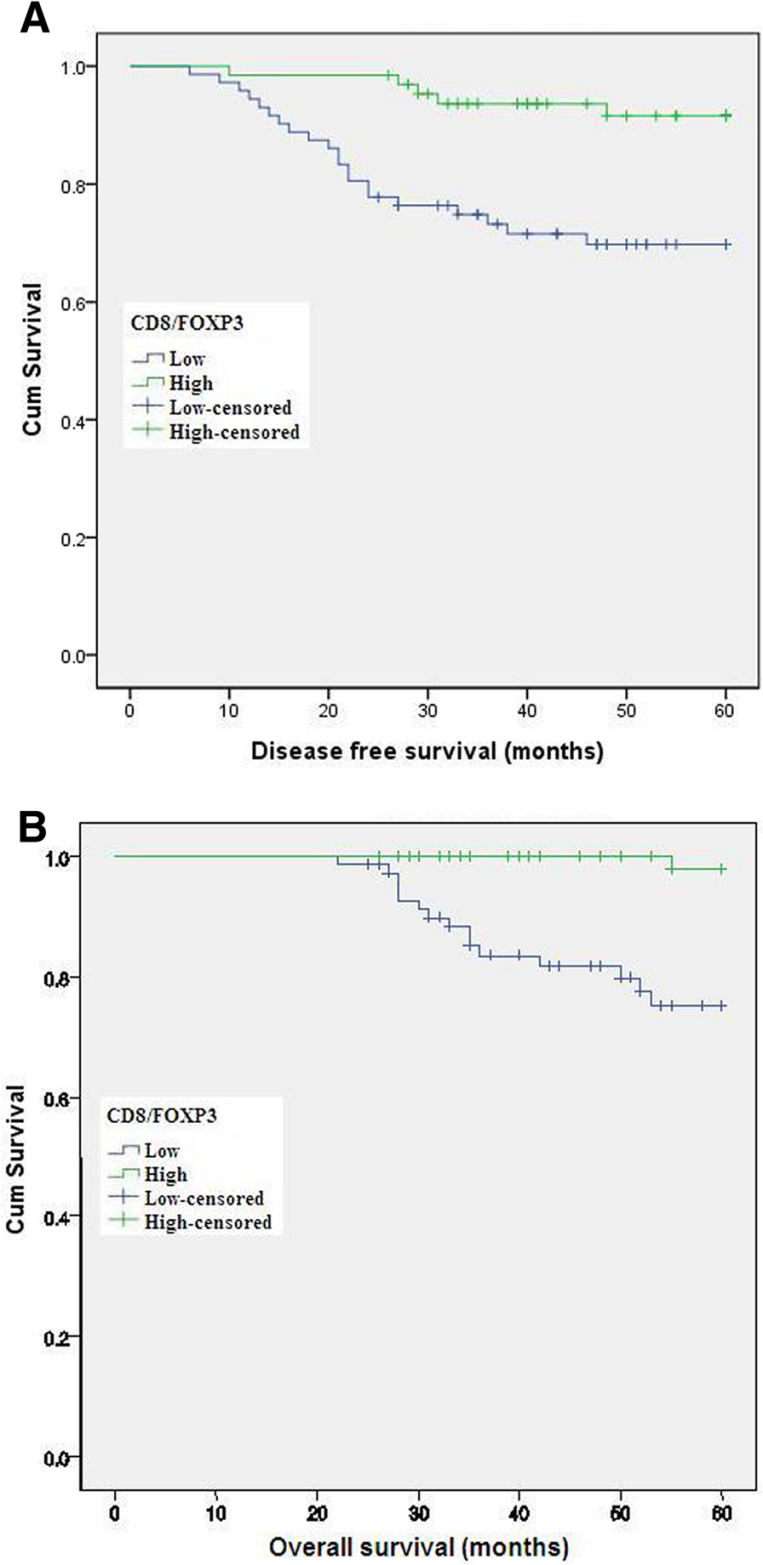
Kaplan-Meier survival curve for patients with high and low CD8/Foxp3 level. The ratio of intratumoral CD8+/peritumoral FOXP3+ cells in residual tumors after NACT was associated with favorable clinical outcomes (**a**: PFS; **b**: OS)

Both univariate and multivariate analyses were performed to determine the association between prognosis and variables, including clinicopathological factors, the levels of CD8+, FOXP3+ T cell and the CD8+/FOXP3+ ratio. In univariate analyses, high intratumoral CD8+ T cell, low peritumoral Foxp3+ T cell as well as the ratio of them were favorable indicators for PFS (*p* = 0.044, *p* = 0.045, *p* = 0.003) and OS (*p* = 0.017, *p* = 0.045, *p* = 0.007) (Table 2). In the multivariate Cox regression model, only the intratumoral CD8+/peritumoral FOXP3+ ratio remained prognostic significance for both PFS (OR: 0.297, 95% CI 0.109–0.810, *p* = 0.018) and OS (OR: 0.078, 95% CI 0.010–0.598, *p* = 0.014) (Table 3, Fig. 4).

**Table 2:** Univariate analysis of clinical parameters and TILs for cervical cancer survival after NAC

	PFS			OS		
	HR	95% CI	*P*	HR	95% CI	*P*
Intratumoral CD8 before NAC	0.823	0.381–1.779	0.620	0.721	0.268–1.936	0.516
Peritumoral CD8 before NAC	*1.040*	*0.482–2.244*	*0.920*	1.424	0.530–3.826	0.483
Intratumoral CD8 after NAC	0.425	0.185–0.979	**0.044**	0.215	0.061–0.756	**0.017**
Peritumoral CD8 after NAC	0.500	0.223–1.123	0.093	0.451	0.157–1.298	0.140
Intratumoral FOXP3 before NAC	1.588	0.717–3.482	0.257	1.645	0.598–4.527	0.335
Peritumoral FOXP3 before NAC	1.265	0.585–2.735	0.550	1.432	0.533–3.848	0.477
Intratumoral FOXP3 after NAC	0..983	0.455–2.120	0.964	1.035	0.388–2.757	0.946
Peritumoral FOXP3 after NAC	2.341	1.017–5.386	**0.045**	3.182	1.024–9.891	**0.045**
Intratumoral CD8/Peritumoral FOXP3 after NAC	0.227	0.085–0.602	**0.003**	0.061	0.008–0.463	**0.007**
OR(OR vs. Non-OR)	0.165	0.022–1.214	**0.077**	0.249	0.0432–1.536	**0.037**
Clinical response (Yes vs. No)	0.419	0.194–0.905	**0.027**	0.397	0.148–1.066	**0.067**
Lymph node metastasis(Yes vs. No)	2.308	0.970–5.493	**0.059**	1.903	0.614–5.902	0.265
Chemotherapy regimen(BVP vs. TP)	1.150	0.512–2.585	0.735	1.668	0.573–4.860	0.348
Histological grade (I-II vs. III)	1.016	0.401–2.574	0.973	1.668	0.579–4.803	0.343
FIGO stage (Ib2 vs. IIa)	2.183	1.011–4.710	**0.047**	2.888	1.074–7.767	**0.036**
Age (< 40 vs. > 40)	1.630	0.561–4.731	0.369	0.974	0.921–1.031	0.368

To bring more possible covariates into multivariate cox regression analysis, a *p*-value less than 0.1 is believed to have significant difference in this univariate regression analysis

**Table 3:** Multivariate analysis of clinical parameters and TILs for cervical cancer survival after NAC

	PFS			OS		
	HR	95% CI	*P*	HR	95% CI	*P*
OR(OR vs. Non-OR)	0.299	0.039–2.298	0.246	0.412	0.065–3.216	0.967
Clinical response (Yes vs. No)	0.514	0.236–1.120	0.094	0.556	0.204–1.513	0.250
FIGO stage (Ib2 vs. IIa)	1.560	0.686–3.551	0.289	2.212	0.808–6.508	0.122
Intratumoral CD8/Peritumoral FOXP3 after NACT	0.297	0.109–0.810	0.018	0.078	0.010–0.598	**0.014**

## Discussion

TILs play an important role in tumor immunity. Tumor-infiltrating CD8+ T cells are believed to be the front fighter against tumor, while Foxp3+ T cells can suppress the proliferation and activation of CD8+ T cells [8]. In this study, we tried to assess the number and distribution of these two TILs in cancer tissues. Early studies did not concern on the lymphocytic subtypes and they found that there was a significant association between a low density of TILs and local disease control failure [9]. Subsequent studies showed that the subtype, number and location of TILs may play different roles in the development of cervical cancer. Piersma etc. found that elevated intraepithelial CD8+ T cells were associated with no lymph node metastases in patients with large early-stage cervical cancer [10]. Foxp3+ T cells increased with the progression of cervical neoplasia [11]. In line with previous studies, we here demonstrated that high intra-tumor CD8+ T cells were negatively associated with lymph node metastasis.

In practice, clinical response was evaluated according to WHO tumor response criteria [6]*.* As the alteration of tumor volume was only available after NACT, a delay of treatment may occur in the patients who are insensitive to chemotherapy. Identifying patients who might be responsive to chemotherapy prior to treatment becomes critical in personalized treatment and outcomes. In this work, we found that the pre-treatment low peritumoral Foxp3+ infiltration is able to screen out those patients who are clinically sensitive to chemotherapy. In other cancers, such as non-small cell lung cancer, investigators have also found a significant correlation between Treg reduction and clinical response to neoadjuvant chemotherapy [12]. All these results support a previous hypothesis that pretreatment host immune states can influence the effectiveness of chemotherapy against cancer cells.

To the best of our knowledge, the current work is the first study to evaluate the dynamic changes of the TIL subsets in the cervical cancer tissue before and after NACT. We found platinum/taxane-based neoadjuvant intervention significantly decreased the frequencies of Foxp3+ T cells as compared to NACT naive biopsies, whereas the CD8+ T cells accumulation remained unaffected. Furthermore, we noted that the diminished Foxp3+ T cells were associated with a high-pCR rate. We speculated that reduction of Foxp3 by chemotherapy may diminish the immunologic suppression, thus resulting in a high pCR rate in cervical cancer with both CD8 and Foxp3 infiltration. The changes of TILs were also seen in other cancers after chemotherapy. In ovarian carcinoma treated with NACT, local CD8+ cells increased while Foxp3+ cells remained stable [13]. In breast cancer, both CD8+ and Foxp3+ TILs increased after NAC, but CD8+ lymphocytes increased more dramatically resulting an elevated CD8+/FOXP3+ ratio [14]. The variable change of TILs in these studies may be due to the different tumor background or cytotoxic agents. However, it is notable that the CD8+/FOXP3+ ratio increased in all these studies which indicate the cytotoxic agents may modulate T cell infiltration irrespective of the tumor background. One study showed that the tumor infiltrating CD8+ T cells decreased after irradiation in cervical cancer while Foxp3+ T cells did not show any alterations, suggestive of the local anti tumor immunity suppression [15]. These findings implied that although chemotherapy and radiation were used to control tumor progression in cervical cancers, the influence on the local immune microenvironment might be different.

We evaluated the predictive value of TILs in residual tumors of cervical cancer patients who underwent NACT. We found that the intratumoral CD8+ T cells, peritumoral Foxp3+ T cells and the ratio of them in residual tumors were associated with survival, but only the CD8+/ Foxp3+ ratio was an independent prognostic factor. These findings suggest that the balance or interplay between CD8+ and Foxp3+ T cells in the tumor microenvironment determine the clinical outcome rather than the absolute number of TILs. Previous studies have addressed the predictive relevance of individual immune cell subpopulations in residual cancers after chemotherapy. Oesophageal adenocarcinoma patients with a high CD8+ T cells density after NACT had a favorable clinical outcome [16], while the presence of Foxp3+ TILs in breast cancer patients predicted a good response to NACT and a favorable prognosis [17]. The discrepancy among these studies may reflect the different tumor background, the diverse methods to calculate the prevalence of TILs subtypes, and the different chemotherapeutic regimens.

In our study, we found the compartmental localization of CD8+ and Foxp3+ cells might influence the impact of tumor immunity and only CD8+ cells in tumor area and Treg+ cells in surrounding stroma had relevance with clinical factors and tumor progression. It is presumable that TILs are activated only in certain locations. In breast cancer, Gobert et al. found that Treg within lymphoid infiltrates surrounding the tumor are selectively recruited through CCL22/CCR4 and activated with high levels of membrane GITR and ICOS expression [18]. The precise mechanisms of activation of TILs in cervical cancer remains unclear and the biological difference between intra- and peri- TILs needs further investigation.

Our work has some limitations. The first limitation is the heterogeneity in adjuvant post-operative therapies, which might affect survival outcome in NACT patients with risk factors. However, several studies reported equivalent therapeutic results between adjuvant chemotherapy, chemoradiotherapy, and radiotherapy after radical surgery [19, 20]. Second, the cut-points of low and high lymphocytic infiltration merit precision definition although lymphocytic infiltration is emerging as a potential prognostic factor in our research. A third limitation inherent to other studies is that the evaluation of certain TILs is probably only an imperfect surrogate of the type of immune response in the tumor microenvironment.

## Conclusions

In conclusion, we evaluated the effects of NACT on local CD8+ and Foxp3+ T cells in cervical cancer. We found a beneficial change in the immunologic balance within the tumor microenvironment after NACT, namely, decreased Foxp3+ T cells and stable CD8+ T cells. Furthermore, low levels of peritumoral Foxp3+ cells before treatment was able to identify a group of patients who were highly sensitive to NACT, while the intratumoral CD8+/ peritumoral FoxP3+ ratio in residual tumors was the predictor of DFS and OS for locally advanced cervical cancer patients.

## Abbreviations

DFS: Disease-free survival; OS: Overall survival
FOV: Field of view
HPV: Human papillomatous virus
NACT: Neoadjuvant chemotherapy
pOR: pathological optimal response
TILs: Tumor infiltrating lymphocytes
WHO: World health organization
